# A Rare Case of Herpes Zoster in an Adult Patient Recovered From Symptomatic Reinfection of COVID-19

**DOI:** 10.7759/cureus.16274

**Published:** 2021-07-08

**Authors:** Binita Ghosh, Rohan A Gajjar, Vaishnavi K Modi, Dhigishaba M Jadeja

**Affiliations:** 1 Internal Medicine, Burdwan Medical College, Burdwan, IND; 2 Internal Medicine, BJ Medical College, Ahmedabad, IND; 3 Internal Medicine, Baroda Medical College, Vadodara, IND; 4 Internal Medicine, Gujarat Adani Institute of Medical Sciences, Bhuj, IND

**Keywords:** herpes zoster, varicella zoster, covid-19, sars-cov-2, adult

## Abstract

In coronavirus disease 2019 (COVID-19) patients, various dermatological conditions have been observed. Varicella zoster virus (VZV) and herpes simplex virus must be ruled out before considering vesicular exanthems linked to COVID-19. The immunological status of the host has an impact on the natural history of herpes zoster (HZ). Age is a major risk factor for most of the cases of HZ. Reactivation of VZV can be triggered by iatrogenic immunosuppression or disease-related immunocompromised state or age-related immunosenescence. Rarely, dermatological symptoms have been reported in recovered COVID-19 patients. We hereby present a rare case of HZ in a recovered patient from symptomatic reinfection of COVID-19.

## Introduction

As of June 8, 2021, the severe acute respiratory syndrome coronavirus 2 (SARS-CoV-2), has affected 29,762,793 people and caused 383,490 deaths, with a case fatality rate of 1.28% in India, which was 1.60% as of September 22, 2020 [1]. The present coronavirus disease 2019 (COVID-19) is a worldwide multisystemic disorder with devastating medical and social implications [2-4]. While COVID-19 related cutaneous symptoms are becoming more common, their precise incidence is yet to be determined, their pathophysiological processes are still unclear, and the involvement of SARS-CoV-2 in their pathogenesis, whether direct or indirect, is still disputed. Herpes zoster (HZ) is one of the frequently reported cutaneous conditions in COVID-19 positive patients. There are only a few cases of HZ reported in the literature following recovery from COVID-19. Prolonged dermatological symptoms in recovered COVID-19 patients are uncommon. We hereby, present a rare case of HZ in a recovered adult patient from symptomatic reinfection of COVID-19. 

## Case presentation

**Figure 1:**
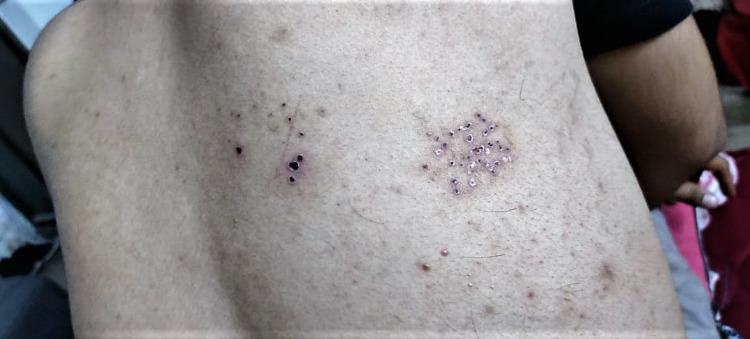
Skin lesion in recovered COVID-19 adult patient. COVID-19, coronavirus disease 2019

A 26-year-old male working as a resident medical officer (RMO) at the tertiary care center presented to the dermatology clinic with the chief complaint of painful blisters with ulcerations and mild itching over thoraco-abdomen corresponding to right T11-12 dermatome. His past medical history was unremarkable except for positive SARS-CoV-2 infection diagnosed through nasal swab reverse transcriptase-polymerase chain reaction (RT-PCR) in May 2020. At that time, he was treated with hydroxychloroquine, azithromycin, and with other supportive treatments. He successfully recovered after one week of treatment. He was deemed negative by the resolution of his COVID-19 symptoms. He again developed symptomatic reinfection of COVID-19 on April 21, 2021, diagnosed through RT-PCR. Although, gene sequencing was not performed. At this time, he was treated with favipiravir, ivermectin, azithromycin, aspirin, vitamin C, and vitamin D. He recovered after two weeks of COVID-19 diagnosis. On May 15, 2021, typical symptoms of HZ started to develop such as vesicles with surrounding erythema, painful blisters with ulcerations, mild itching, and unilaterally characterized to the thoraco-abdomen region corresponding to right T11-12 dermatome resulting in a clinical diagnosis of HZ. Furthermore, he had a history of VZV infection in his childhood. At the time of the appearance of HZ-specific symptoms, his RT-PCR for SARS-CoV-2 came negative. Furthermore, he was tested for human immunodeficiency virus (HIV) antibodies in July 2020, which came non-reactive. Laboratory blood markers such as total white blood cells (WBC) and absolute lymphocyte count (ALC) were within the normal range (Figure 1).

He was successfully treated with acyclovir 800 mg five times a day orally with other supportive symptomatic treatments for eight days. On follow-up evaluation, cutaneous manifestation has resolved and was continued on supportive treatment with multivitamins. 

## Discussion

COVID-19 disease involves multiple organ systems in the human body causing respiratory, cardiovascular, renal, neurological, hematological, olfactory, gustatory, ocular, cutaneous, and gastrointestinal manifestations [5-6]. Contemporary studies revealed different dermatological symptoms in patients with COVID-19 including maculopapular erythematous rash, vesicles, urticaria, chilblains-like lesions (COVID toes), vasculopathic lesions like petechiae, livedo reticularis, cyanosis in finger and toes, skin bullae, and dry gangrene [7]. Although most of the cutaneous manifestations of COVID-19 have been attributed to microthromboses, lymphocytic vasculitis, or Langerhans cell activation by the SARS-CoV-2 [8]. A few studies described an increase in HZ co-infection causing skin lesions during the COVID-19 pandemic [9]. HZ is caused by the reactivation of latent varicella virus in dorsal root ganglia years after primary varicella infection. The reactivated virus travels along sensory axons to infect epithelial cells causing vesicular skin rash within the dermatome innervated by the particular sensory nerve. The reactivation of the VZV is considered to be a result of a decline in VZV-specific cell-mediated immunity most commonly due to aging or other causes of immunosuppression. In immunocompetent adults, HZ is usually seen in the age group above 50 years [9]. Similarly, the majority of the cases of HZ developed in patients with COVID-19 were reported in patients aged above 50 years [9]. However, Nofal et al. described four immunocompetent patients developing HZ ophthalmicus in the age group of 7-42 years during active COVID-19 infection [10]. Most of the cases of HZ in COVID-19 patients developed skin lesions in a period ranging from two days before to seven days after onset of COVID-19 symptoms [10-13]. Studies have reported the development of HZ following recovery of COVID-19 [7, 14].

Reactivation of HZ in COVID-19 patients can be explained by the immune alteration apparent in such patients. A decrease in total lymphocytes, a cluster of differentiation (CD4+) T cells, CD8+ T cells, B cells, and natural killer (NK) cells are observed in COVID-19 patients [9]. Functional exhaustion of CD8+ T cells and NK cells with increased expression of natural killer cell receptor (NKG2A) causing decreased antiviral immunity is also evident in the early stages of SARS-CoV-2 infection [15]. 

We report a case of HZ in a 26-year-old male working as RMO two weeks after recovery from COVID-19. He presented with a painful vesicular rash in T11-T12 dermatomes. Unlike most of the other cases reported, our patient developed symptoms of HZ at a relatively younger age and few weeks after recovery from the symptomatic reinfection of COVID-19. Literature is now buzzing with symptomatic reinfection of COVID-19 [16]. His recovery was confirmed with a negative RT-PCR test for symptomatic reinfection of SARS-CoV-2, lack of immunoglobulin (IgM), and presence of IgG antibodies against SARS-CoV-2. WBC count was found to be normal at the time of onset of HZ. Various studies have reported psychological stress as a risk factor for the development of HZ [17-19]. In the present case, stresses related to his hectic in-hospital COVID-19 emergency duty, post-COVID stress disorder may have served as a mechanism for the development of HZ following recovery from COVID-19. Furthermore, due to mandated social distance and a lack of viable therapies during the COVID-19 pandemic, telemedicine has proven to be the safest interaction method between patients and physicians [20]. Many skin conditions can be diagnosed and prevented through telemedicine during such pandemics. 

## Conclusions

The majority of the cases of HZ developed in patients with COVID-19 were reported in patients aged above 50 years. To date, few cases reported dermatological manifestation following recovery from COVID-19. To our knowledge, this is the first case of HZ developed in an adult patient following recovery from COVID-19. Nevertheless, the number of total lymphocytes, T cells, B cells, and NK cells increased upon recovery from the disease. Further studies are required to explore the factors that predispose patients to the development of HZ following recovery from COVID-19 particularly in patients who are at developing post-COVID stress disorder.

## References

[1] Ansari AA, Desai HD, Sharma K, Jadeja DM, Patel R, Patel Y, Desai HM (2021). Prevalence and cross states comparison of case fatality rate and recovery rate of COVID 19/SARS-COV-2 in India. J Family Med Prim Care.

[2] Desai HD, Sharma K, Jadeja DM, Desai HM, Moliya P (2020). COVID-19 pandemic induced stress cardiomyopathy: a literature review. Int J Cardiol Heart Vasc.

[3] Desai HD, Jadeja DM, Sharma K (2020). Takotsubo syndrome a rare entity in patients with COVID-19: an updated review of case-reports and case-series. Int J Cardiol Heart Vasc.

[4] Singhavi R, Sharma K, Desai HD, Patel R, Jadeja D (2020). A case of hemolytic anemia with acute myocarditis and cardiogenic shock: a rare presentation of COVID-19. Cureus.

[5] Desai HD, Sharma K, Parikh A (2021). Predictors of mortality amongst tocilizumab administered COVID-19 Asian Indians: a predictive study from a tertiary care centre. Cureus.

[6] Sharma K, Desai HD, Patoliya JV, Jadeja DM, Gadhiya D (2021). Takotsubo syndrome a rare entity in COVID-19: a systemic review-focus on biomarkers, imaging, treatment, and outcome. SN Compr Clin Med.

[7] Desai HD, Sharma K, Patoliya JV, Ahadov E, Patel NN (2021). A rare case of Varicella-Zoster virus reactivation following recovery from COVID-19. Cureus.

[8] Sachdeva M, Gianotti R, Shah M (2020). Cutaneous manifestations of COVID-19: report of three cases and a review of literature. J Dermatol Sci.

[9] Diez-Domingo J, Parikh R, Bhavsar AB, Cisneros E, McCormick N, Lecrenier N (2021). Can COVID-19 increase the risk of herpes zoster? A narrative review. Dermatol Ther (Heidelb).

[10] Nofal A, Fawzy MM, Sharaf El Deen SM, El-Hawary EE (2020). Herpes zoster ophthalmicus in COVID-19 patients. Int J Dermatol.

[11] Tartari F, Spadotto A, Zengarini C (2020). Herpes zoster in COVID-19-positive patients. Int J Dermatol.

[12] Saati A, Al-Husayni F, Malibari AA, Bogari AA, Alharbi M (2020). Herpes zoster co-infection in an immunocompetent patient with COVID-19. Cureus.

[13] Shors AR (2020). Herpes zoster and severe acute herpetic neuralgia as a complication of COVID-19 infection. JAAD Case Rep.

[14] Brambilla L, Maronese CA, Tourlaki A, Veraldi S (2020). Herpes zoster following COVID-19: a report of three cases. Eur J Dermatol.

[15] Zheng M, Gao Y, Wang G (2020). Functional exhaustion of antiviral lymphocytes in COVID-19 patients. Cell Mol Immunol.

[16] Vora T, Vora P, Vora F, Sharma K, Desai HD (2021). Symptomatic reinfection with COVID-19: a first from Western India. J Family Med Prim Care.

[17] Schmader K, Studenski S, MacMillan J, Grufferman S, Cohen HJ (1990). Are stressful life events risk factors for herpes zoster?. J Am Geriatr Soc.

[18] Schmader K, George LK, Burchett BM, Pieper CF (1998). Racial and psychosocial risk factors for herpes zoster in the elderly. J Infect Dis.

[19] Schmader K, George LK, Burchett BM, Hamilton JD, Pieper CF (1998). Race and stress in the incidence of herpes zoster in older adults. J Am Geriatr Soc.

[20] Sharma K, Desai HD (2021). Role of self-measured home blood pressure monitoring (HBPM) and effectiveness of telemedicine during the era of COVID-19 pandemic. SN Compr Clin Med.

